# Specific for Singultus

**Published:** 1883-08

**Authors:** 


### SPECIFIC FOR SINGULTUS.

This very common affection, of infants and children especially, has a specific remedy, at least one which I have never known to fail. Moisten granulated sugar with good cider vinegar; give to an infant from a few grains to a teaspoonful.

The effect is almost instantaneous, and the dose seldom needs to be repeated.

I have used it from all ages, from infants a few months old, to those on the down hill side of life. Try it.—*Henry Tucker, M.D., Brattleboro, Vt.*

We remember in our first year of practice having been called, in consultation, to see a very ill man with hiccough; after having tried valerian, prussic acid, morphia, and other agents, without avail, the atttending physician said to us: "Doctor, I can tell you what will stop this hiccough, if you will promise not to laugh at the remedy." We assured him that we would be very grave under the circumstances and should like to be taught. He accordingly returned to the sick room and asked, to my astonishment, if they had any *damson preserves* about the house?

After much hunting some were obtained from a neighbor and a spoonful or two duly administered to the wondering patient, who was relieved very speedily. I have tried it on many occasions with excellent results, and suppose that the sugar is the agent that acts so well, or it may be the damson.

I generally give the *original recipe* in obstinate cases, however.—*Ed. So. Clinic.*

We would append to this addendum of the editor of *Southern Clinic*, a reference to a most remarkable case of hiccough, which occurred a few years ago in this city, in the practice of Dr. A. W. Calhoun. We first heard an account of it from the doctor himself. There are only two others who in the telling of it can approximate its merits—Drs. J. G. Westmoreland and J. Thad. Johnson.

A good time, and no little valuable instruction awaits the man who, after a good dinner for all concerned, and loosened suspenders, shall call upon anyone or all of the above named gentlemen to describe this case and tell just how hiccough ought *not* to be treated.—Ed. Register.

